# Asthma in pregnancy: association of asthma control test (ACT) with clinical management by the global initiative for asthma (GINA)

**DOI:** 10.1186/1939-4551-8-S1-A90

**Published:** 2015-04-08

**Authors:** Georgia Araujo, Guilherme Alves, Bianca Santos, Debora Leite, Mauro Aguiar, Décio Medeiros, José Ângelo Rizzo, Emanuel Sarinho

**Affiliations:** 1 Federal University of Pernambuco, Brazil

## Background

Asthma is probably the most common chronic medical condition in pregnancy, with a worldwide prevalence estimated between 8-13%. The present study aims to identify a possible association between clinical asthma control by the Global Initiative for Asthma (GINA) with ACT at the end of the second trimester of pregnancy, a period considered the highest risk of clinical deterioration and exacerbations of asthma.

## Methods

A retrospective, cross-sectional study of 103 pregnant women with asthma who were treated during the period October 2010 to October 2013, the asthma clinic in pregnancy, Hospital das Clinicas, Federal University of Pernambuco. The level of asthma control by GINA and the ACT for pregnant women with asthma was evaluated in the same time period, between the twenty-first and twenty-seventh week of pregnancy. The association between the ACT and the level of clinical control by GINA by Chi-Square test was performed.

## Results

A total of 103 pregnant patients with asthma, 79 (76,7%) with the age range between 18-34 years of age were analyzed. Analyzing the initial care of these patients, 50 (48,5%) of them were aged < 20 weeks already showing a high body mass index, overweight in 50 (48,5%) of them. Of asthmatic pregnant women, 62 (60,2%) used beta-agonist inhaled short acting during exacerbations and kept using their inhaled corticosteroids continuously with doses of 400 to 800μg per day. Among the most frequent comorbidities, stood out hypertension and gestational diabetes, and among other atopic diseases diagnosed 47 (45,6%) had rhinitis. Mild persistent asthma was more frequent 51 (49,5%) and the presence of asthma control showed 8 (11,2%) uncontrolled, 32 (44,4%) partially controlled and 32 (44,4%) controlled. In the analysis of the association chi-squared test was used and statistically significant association between the clinical control of asthma by GINA and the ACT in pregnancy with p < 0.001.

## Conclusions

This study shows that both clinical control by GINA as ACT can be used in asthmatic pregnant women, especially at the end of the second trimester, a period of deterioration and exacerbations of asthma in pregnancy. We include the importance of the ACT to be a subjective instrument of easy application, interpretation and with good reproducibility that does not require spirometry to assess the level of asthma control in pregnancy.

